# Reflectometric-based sensor arrays for the screening of kinase-inhibitor interactions and kinetic determination

**DOI:** 10.1007/s00216-025-05770-x

**Published:** 2025-02-10

**Authors:** Viola Wurster, Peter Fechner, Günther Proll, Rebecca Pamies-Cuberos, Matthias Frech, Jens Baumgärtner, Antonia Malissa, Martina Marchetti-Deschmann, Natalia P. Ivleva, Christoph Saal, Sebastian Fuchs, Sébastien Moniot, Anja Göttsche, Carolin Huhn

**Affiliations:** 1 Institute of Physical and Theoretical Chemistry, Eberhard Karls Universität Tübingen, Auf der Morgenstelle 18, Tübingen, 72076 Germany; 2 Faculty of Life Science, Reutlingen University, Alteburgstraße 150, Reutlingen, 72762 Germany; 3 Merck KGaA, Frankfurter Straße 250, Darmstadt, 64293 Germany; 4 Institute of Chemical Technologies and Analytics, Technical University of Vienna, Getreidemarkt 9, Vienna, 1060 Austria; 5 Institute of Water Chemistry, Chair of Analytical Chemistry and Water Chemistry, Technical University of Munich, Lichtenbergstraße 4, Garching, 85748 Germany; 6 Present Address: Boehringer-Ingelheim Pharma GmbH & Co. KG, Birkendorfer Straße 65, 88397 Biberach, Germany

**Keywords:** Reflectometric interference spectroscopy, 1-Lambda reflectometry, Staurosporine, Focal adhesion kinase, Spotting, Binding kinetics

## Abstract

Kinases are involved in numerous cellular processes but possibly also in tumor progression. Several kinase inhibitors are approved as drugs and there is an intense search for new inhibitors in pharmaceutical research. In this study, we present a new analytical method based on reflectometric interference spectroscopy, RIfS, for kinase and inhibitor screening. First, the sensor surface was optimized to reduce non-specific binding. Different inhibitors, e.g. staurosporine or fasudil, were immobilized on the transducer surface. Different kinases (focal adhesion kinase and cAMP-dependent protein kinase) were flushed over the sensor with the immobilized inhibitors. The specific interaction was proven by binding inhibition assays. The kinase-inhibitor interaction was monitored label-free and recorded in real time allowing the binding curves to be used to determine the association and dissociation rate constants as well as the affinity. These constants differed depending on the specific kinase-inhibitor pair, which was well expected from parallel docking simulations and measurements with microscale thermophoresis. The strategy was successfully transferred to 1-lambda reflectometry, a modification of RIfS, to enable the simultaneous monitoring of several kinase-inhibitor interactions in 5×7 small spots increasing throughput and automation on a sensor array with imaging detection. Importantly, the techniques developed here can provide both kinetic and thermodynamic data for a multitude of kinases in a single screening approach, which allows for both protein kinase and inhibitor screening.

**Graphical Abstract:**

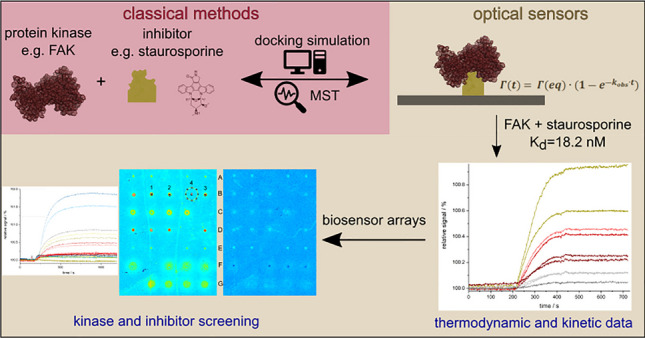

**Supplementary Information:**

The online version contains supplementary material available at 10.1007/s00216-025-05770-x.

## Introduction

As cancer is the second most deadly disease worldwide and the primary cause of death for people younger than 85 years, the search for anti-tumor drugs is intense [1]. The number of cancer patients increases each year [2]. In carcinogenesis, protein kinases have a huge influence. They promote vital cellular processes such as proliferation and cell growth by phosphorylating enzymes to regulate their activity (using adenosine triphosphate, ATP) [3]. As this process is non-selective, both healthy and tumor cells are affected, making common serine/threonine or tyrosine kinases interesting drug targets [4, 5]. The goal is to identify inhibitors that target the ATP-binding pocket of specific protein kinases to inhibit protein kinase phosphorylation [6] and thus specific cellular processes. Inhibitors are characterized by their binding affinity (thermodynamics) and their binding kinetics (on- and off-rate). However, many analytical methods commonly applied only provide thermodynamic data [7]. As a third aspect, the specificity of inhibitor-kinase interactions is relevant, which should be addressed using screening methods providing both kinase and inhibitor screening to reduce analysis time and costs [8, 9]. The ideal analytical method would thus enable kinase and inhibitor screening in combination with the determination of kinetic and thermodynamic data of the binding process.

For this, label-free methods are advantageous as they avoid labeling of one of the binding partners which possibly influences the interaction and represents an additional step including additional work and additional compounds. Separation techniques combined with mass spectrometry and many sensor assays can be used. Among them, biosensors can also be used to obtain kinetic data through real-time monitoring [7]. Reflectometric interference spectroscopy (RIfS) measures changes in the optical thickness (product of physical thickness and refractive index) upon binding when one binding partner is flushed over a sensor surface with the other binding partner being immobilized [10]. Being a spectroscopic technique, RIfS provides detailed information on the binding process. A variant of RIfS, 1-lambda reflectometry, can screen interactions with the possibility to immobilize different proteins or ligands in small spots in an array format via imaging detection [11, 12]. With the possibility for the screening applications, 1-lambda reflectometry provides a higher throughput compared to RIfS.

In this study, we apply RIfS and 1-lambda reflectometry for the first time to investigate kinase-inhibitor interactions. Staurosporine (STP) is employed as a model inhibitor [13, 14] as it interacts competitively with ATP in the ATP-binding pocket of many serine/threonine and tyrosine kinases [13, 15–17]. Additionally, two approved drugs, imatinib (the first kinase inhibitor approved as a drug) and fasudil, as well as two promising molecule fragments [18], were used to demonstrate screening possibilities [19, 20]. The tyrosine kinase focal adhesion kinase (FAK) and a protein mixture containing the serine/threonine kinase cAMP-dependent protein kinase (PKA) were chosen [21]. In tumor cells, FAK was found to be overexpressed and is a highly significant drug target in pharmaceutical research [22–24]. PKA was the first protein kinase to be crystallized and has been extensively studied so that detailed mechanistic information for numerous cellular processes is available [25–28].

## Materials and methods

### Chemicals

Acetone, bovine serum albumin, dichloromethane (DCM), dimethylformamide (DMF), dimethyl sulfoxide (DMSO), ethanol, glycine, guanidine hydrochloride, human serum albumin, hydrogen peroxide, *N*-hydroxy succinimide (NHS), 2-(*N*-morpholino)ethanesulfonic acid (MES), ovalbumin, potassium dihydrogen phosphate (KH_2_PO_4_), sodium acetate, sodium chloride, sodium dodecyl sulfate, sinapinic acid, sulfuric acid, SuperBlock Blocking buffer in PBS, TRIS, trypsin-EDTA, 1-ethyl-3-(3-dimethylaminopropyl) carbodiimide (EDC), (3-glycidylpropyl) trimethoxysilane (GOPTS), Tween 20, and urea were purchased from Sigma-Aldrich, Darmstadt, Germany. Aminodextran was purchased from Innovent, Jena, Germany. Diisopropylcarbodiimide (DIC) and glutaric anhydride (GA) were purchased from Fluka, Buchs, Switzerland. Magnesium chloride was purchased from Thermo Fisher Scientific, Vilnius, Lithuania. Diaminopolyethylene glycol (Da-PEG) (2 kDa) was purchased from Rapp Polymere, Tübingen, Germany.

#### Buffers and solutions

The TRIS buffer, used for diluting the protein kinases and inhibitors, contained 20 mM TRIS and 10 mM MgCl_2_, adjusted to pH 7.4. MES buffer at a concentration of 0.5 M was adjusted to pH 6.3. For RIfS and 1-lambda measurements, the TRIS buffer contained 1% v/v DMSO and was flushed over the sensor to obtain all base- and endlines, to initiate the dissociation phase and used as solvent for the sample solutions. Piranha etching solution was made from sulfuric acid and hydrogen peroxide (both concentrated) in a volume ratio of 3:1.

#### Protein kinases

The protein mix contained cAMP-dependent protein kinase, PKA (0.5 units/µg protein), from bovine heart together with a large number of other bovine heart proteins such as ATP synthase and heat shock protein (as determined by LC-MS analysis during this study) and was purchased from Sigma-Aldrich, Steinheim, Germany. The fragment of focal adhesion kinase 410–689 (FAK) was purified by Merck, Darmstadt, Germany. For MST experiments, further kinase fragments were used, all provided by Merck, Darmstadt, Germany: cAMP-dependent spleen tyrosine kinase 356–635 (SYK), transforming growth factor beta 200–503 (TGFβ), and protein kinase B 144–445 (PKB).

#### Inhibitors

**Fig. 1:**
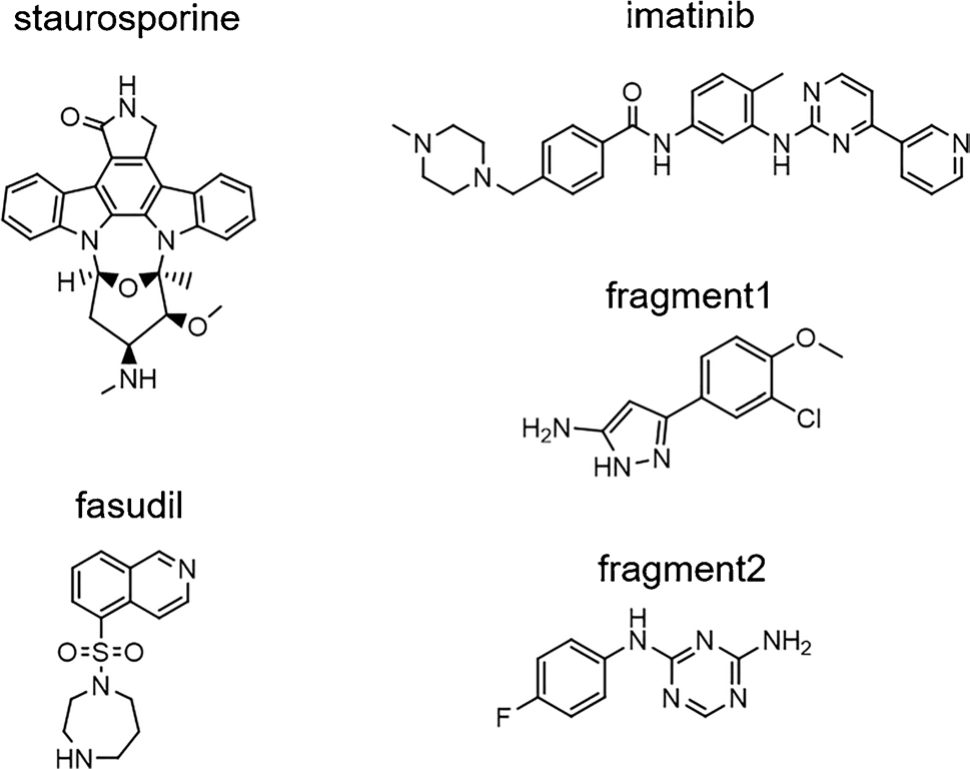
Molecular structures of the small molecules utilized as inhibitors in this work

Staurosporine (STP) was purchased from Alfa Aesar, Thermo Fisher, Kandel, Germany. Fluorescently labeled STP was purchased from Perkin Elmer, MA, USA. Fasudil and imatinib were purchased from Sigma-Aldrich, Steinheim, Germany. Fragment1 and fragment2 were provided by Merck, Darmstadt, Germany. Inhibitor structures are shown in Fig. 1.

### Instrumentation

Transducers for the reflectometric interference spectroscopy (RIfS) consisted of a glass substrate with a Ta_2_O_5_ layer (10 nm) followed by a SiO_2_ layer (330 nm) from Schott, Mainz, Germany. In 1-lambda reflectometry, anti-reflection glass transducers were used.

Both sensor setups included a pump system (Hamilton injection fluidics, Microlab 500 series, double injection dispenser (2×100 µL) Hamilton Company, Reno Nevada, USA) combined with a valve with different inlets for samples (Hamilton-6-way-outlet, Microlab MVP, Hamilton Company, Reno Nevada, USA). The buffer, sample, and regeneration solutions were flushed over the modified transducer surface via optimized microfluidics. In RIfS, white light was irradiated to the backside of the transducer, and the reflected light was guided to the spectrometer (SPEKOL 1100, Analytik Jena, Jena, Germany) via a y-shaped optical fiber. The evaluation of the interference spectra was performed with the software “IFZ and Measure” developed by the working group of Prof. Gauglitz (Eberhard Karls Universität Tübingen, Germany [29, 30]). In 1-lambda reflectometry, green light (535 nm) is irradiated from an LED to the backside of the transducer in a self-constructed flow cell described by Wurster [31]. Images of the transducer were recorded by a CCD camera (pco.camera, Excelitas Technologies, Waltham, MA, USA). The binding of kinases leads to changes in the thickness of the surface layer on the sensor during the measurement, which is reflected by changes in the intensity of the green light detected by the camera. The intensity changes in these images during a measurement were evaluated with ImageJ (US National Institutes of Health, Bethesda, Maryland, USA) as in previous studies [32].

Spotting was performed with the MicroGrid II (BioRobotics, MA, USA) using Xtend capillary microarray pins (LabNEXT, Wolfville, Canada) and the TAS Application Suite (BioRobotics, MA, USA).

### Surface chemistry

The transducers were activated with piranha etching solution for 15 min in an ultrasonic bath, washed with milliQ water, and dried under a stream of nitrogen. Activated transducers were silanized with GOPTS (10 µL for RIfS transducers, 100 µL for 1-lambda reflectometry transducers) in a sandwich technique for 1 h in a dry chamber at ambient temperature. Then, the transducers were washed with water-free acetone and dried under a stream of nitrogen. To reduce non-specific interactions, we immobilized aminodextran or polyethylene glycol as biopolymers on the transducer surface using a sandwich technique: aminodextran was dissolved in milliQ water (7 g/mL, 10 µL for RIfS transducers, 100 µL for 1-lambda reflectometry transducers) and immobilized in a chamber with water vapor at ambient temperature. Diaminopolyethylene glycol was dissolved in dichloromethane (DCM, 4 mg/mL, 10 µL for RIfS transducers) and immobilized in a DCM vapor chamber at 70°C. The reaction was allowed to proceed overnight. The surfaces were washed with milliQ water and dried under a stream of nitrogen. The biopolymers on the sensor surface were refunctionalized with glutaric anhydride in dimethylformamide (DMF, 2 g/mL, 10 µL for RIfS transducers, 100 µL for 1-lambda reflectometry transducers) in a sandwich technique for 6 h in a DMF atmosphere at ambient temperature. After washing with milliQ water and drying under a stream of nitrogen, the surfaces were activated via an NHS DIC coupling protocol: NHS (0.15 g/mL) and DIC (0.23 mL/mL) were mixed with DMF and incubated on the surfaces in the sandwich technique (10 µL for RIfS transducers, 100 µL for 1-lambda reflectometry transducers) in a DMF vapor chamber for 4 h at ambient temperature. After this activation, the transducers were washed with water-free acetone and dried again. Inhibitors were then immobilized via their amino groups in a DMSO solution (1 mg/mL) for 1 h at ambient temperature by incubation at 4°C overnight under a DMSO atmosphere. These final transducers were washed with milliQ water, dried under a stream of nitrogen, and stored at 4°C until use.

Spotting: For imaging, the inhibitors were spotted on the refunctionalized and activated transducer surfaces in two ways:

1. Manual spotting: the inhibitor dissolved in DMSO (1 mg/mL) was dropped to the measurement region on the transducer by pipetting. The immobilization process was as specified above.
2. Automatic spotting: the inhibitor dissolved in DMSO (1 mg/mL) was dropped to the measurement region on the transducer via the ceramic pin of the spotter. The droplets were dried under ambient conditions, and the process was repeated twice. After drying the last droplet, the transducer was prepared for measurements or stored at 4°C.

### Protocols for sensor measurements

#### Single assays

Single assays were conducted in RIfS and 1-lambda reflectometry with transducers to which only one inhibitor was immobilized over the complete surface. First, a blocking solution (SuperBlock Blocking buffer) was flushed over the sensor to saturate remaining non-specific binding sites on the sensor (100 µL in 200 s for RIfS, 400 µL in 200 s for 1-lambda reflectometry). The larger flow cell of 1-lambda reflectometry required a larger sensor area and larger sample volumes. After a baseline with TRIS buffer containing 1% DMSO, named “buffer” in the following, was recorded (flushing with 100 µL in 200 s for RIfS, 400 µL in 200 s for 1-lambda reflectometry), the sample was flushed over the sensor for the association phase (100 µL in 200 s for RIfS, 400 µL in 200 s for 1-lambda reflectometry), followed by flushing buffer for the dissociation phase (100 µL in 200 s for RIfS, 400 µL in 200 s for 1-lambda reflectometry). The surface was tried to be regenerated by flushing with various solutions (as described in the Electronic Supplementary Material, Section S2) (500 µL each in 100 s), and finally, buffer was flushed over the sensor to obtain the endline and define the quality of the regeneration process (100 µL for RIfS, 400 µL for 1-lambda reflectometry).

In the titration experiments, all samples were measured in triplicates in RIfS, except for the highest concentration of 100 µg/mL. As the investigations with 1-lambda reflectometry were performed to validate the applicability of the investigations with this detection setup, only selected samples were measured in duplicates, to assure the reproducibility while reducing kinase consumption.

#### Screening

Screening was performed with 1-lambda reflectometry and transducers on which different inhibitors were spotted in the measurement area. The measurement protocols were the same as for the single assays.

#### Kinetic evaluation

Binding kinetics were evaluated by an exponential fit of the curvature of the association phase in the sensograms using the software Origin 2020 (OriginLab, MA, USA) Eq. 1, as described in detail by Fechner et al. [33].

1 $$\boldsymbol{\Gamma }\left({\varvec{t}}\right)=\boldsymbol{ }\boldsymbol{\Gamma }({\varvec{e}}{\varvec{q}})\cdot (1-{{\varvec{e}}}^{{-{\varvec{k}}}_{{\varvec{o}}{\varvec{b}}{\varvec{s}}}\cdot {\varvec{t}}})$$

The equation describes the binding of the kinase to the surface with the immobilized inhibitor via a pseudo-first order kinetic with the surface loading Γ(t) depending on the surface loading under equilibrium conditions Γ(eq) and the observable rate constant *k*_obs_.

The calculated observable rate constant (*k*_obs_) for each sensogram was then plotted against the concentration of the sample. With the linear regression in Eq. 2, the association and dissociation rate constants (*k*_*a*_ [L mol^−1^ s^−1^] and *k*_*d*_ [s^−1^]) were determined:

2 $${{\varvec{k}}}_{{\varvec{o}}{\varvec{b}}{\varvec{s}}}={\varvec{c}}\left({\varvec{s}}{\varvec{a}}{\varvec{m}}{\varvec{p}}{\varvec{l}}{\varvec{e}}\right)\cdot {{\varvec{k}}}_{{\varvec{a}}}+{{\varvec{k}}}_{{\varvec{d}}}$$

Using Eq. 3 with the ratio *k*_*d*_/*k*_*a*_, the affinity or dissociation constant *K*_*d*_ was calculated.

3 $${{\varvec{K}}}_{{\varvec{d}}}=\frac{{{\varvec{k}}}_{{\varvec{d}}}}{{{\varvec{k}}}_{{\varvec{a}}}}$$

### Microscale thermophoresis

Microscale thermophoresis was performed with the instrument Monolith NT.115, capillaries and MO.Control Software from Nanotemper Technologies, Munich, Germany. For each experiment, 16 capillaries were filled with a sample solution. In the direct assays, fluorescently labeled STP (40 nM) (STP-Red, Perkin Elmer, MA, USA) was titrated with kinase, while for every new capillary, half the kinase concentration was used. The fluorescence of a specific area of each capillary was measured prior and during the thermophoresis experiment. For a better comparability, the fluorescence intensity obtained during the measurement was normalized to the fluorescence in each capillary recorded prior to the start of the experiment. The normalized fluorescence recorded from each capillary was displayed in a logarithmic form. In the competitive assays, a complex of the labeled STP and the protein kinase was titrated with another inhibitor.

## Results and discussion

### Optimization of the sensor assay

During the transducer modification, a layer of biopolymer was built to prevent non-specific interactions on the surface and to increase the distance between the surface and the immobilized inhibitor to reduce steric hindrance during binding. Two biopolymers, AMD and diamino-PEG, were tested. While AMD forms a three-dimensional layer and provides a high number of binding sites for the inhibitor, the PEG-matrix is regarded two-dimensional in shape, thus creating only a monolayer of bound inhibitors on the sensor surface [34]. In our study, AMD provided a higher surface capacity for the interactions with the protein sample, which resulted in a higher signal from the sensors. Thus, AMD was used as the biopolymer for all further measurements.

#### Reduction of non-specific binding

To assure the specificity of the binding between the sensor surface and the protein kinases, non-specific interactions on the sensor surface must be prevented. Non-specific interactions were not relevant as demonstrated with measurements with concentrated (1 M) protein solutions containing bovine serum albumin, human serum albumin, and ovalbumin, which were flushed over the transducers modified with only AMD or STP coupled to AMD. No changes in the optical thickness were recorded by RIfS.

#### Regeneration of the sensor surfaces

Early measurements in the study showed difficulties with the regeneration of the sensor surface preventing a re-use of the sensor for further measurements. Unfortunately, all attempts to break the interactions of the kinases with the inhibitors bound to the sensor surface while keeping the surface suitable for further measurements failed and no regeneration was possible. We first tested classical regeneration media [35–38] such as guanidine hydrochloride and urea as chaotropes, sodium dodecyl sulfate, and Tween 20 as detergents, diverse acids and bases, salt solutions with MgCl_2_, or sodium acetate and solutions containing glycine or ethanol. However, all these regeneration media failed in regenerating our transducer surfaces; for details, see Section S2.1.

In a second strategy, the cocktail protocol from Anderson et al., which was developed for the regeneration of sensors used for surface plasmon resonance, was followed [35]; see Section S2.1. Again, no satisfying regeneration was reached. If a regeneration medium was successful, a subsequent new association of protein kinase on the same transducer after this regeneration step was impaired.

In a third strategy, we tried to tryptically digest the kinases, followed by a regeneration step with guanidine hydrochloride. But even this strategy was unsuccessful.

Due to the failure of a complete regeneration, a new transducer was prepared for every measurement. The disadvantage was that the required complex preparation protocol led to slight changes in the transducer surface. These affected the repeatability of measurements visible in differences in the change of the optical thickness with relative standard deviations up to 24%. This has to be reduced in the future to increase the precision in the determination of kinetic data.

The optical thickness did not change upon injection of kinase without inhibitors immobilized to the transducer; thus, we expect the low regeneration efficiency to be caused by an unexpectedly strong interactions between the protein kinases and the inhibitors on the transducers. We hypothesize that more than one interaction site per kinase was involved in the binding, which increased the overall binding strength. According to docking simulations (see Section S3), interaction sites of the inhibitors outside the ATP-binding pocket of the protein kinases are indeed possible, further supported by the assumption of a dense inhibitor surface loading on the sensor. To corroborate this hypothesis, we simulated such an excess of inhibitors in microscale thermophoresis (MST) experiments with the protein kinase being titrated to fluorescently labeled STP (see Section S4). These experiments and also competitive assays in MST, where labeled STP in complex with protein kinase held at a constant concentration was titrated with another inhibitor, showed the possibility of interactions outside the ATP-binding pocket.

In order to better understand the strong binding, we used different surface analytic methods: MALDI-TOF-MS, Raman microscopy, and ATR-IR (see Section S2.2). Unfortunately, all these methods failed in monitoring the expected monolayer of protein attached to the surface after RIfS measurements.

**Fig. 2:**
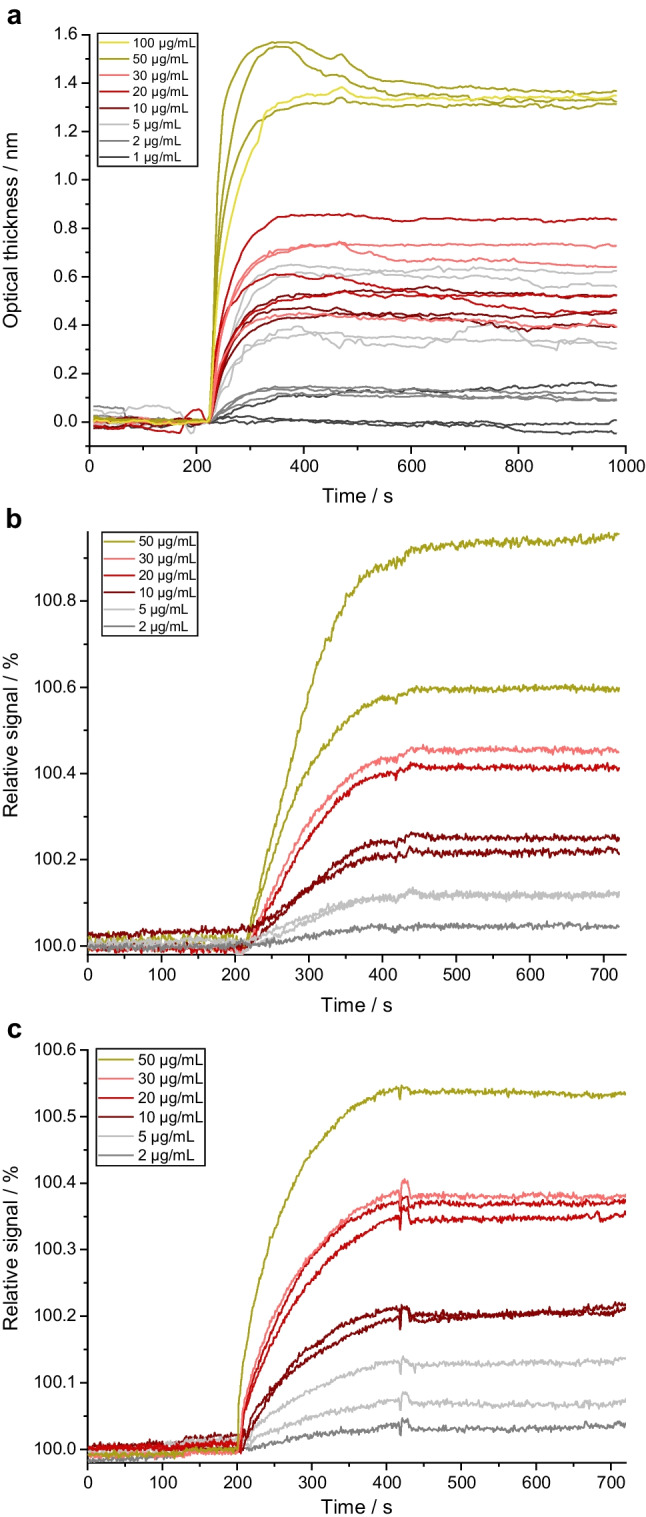
Sensograms of single assays performed with a new transducer each. Different proteins were used over a wide concentration range: **a** FAK-solutions (1–100 µg/mL), **c**, **b** protein mix solution (2–50 µg/mL). The inhibitors immobilized on the surface were **a**, **b** STP, **c** fragment1. The analysis was made using **a** RIfS and **b**, **c** 1-lambda reflectometry. Analyses were made in different replicates: **a** 4 replicates for 5 mg/mL, 3 replicates for 1, 2, 20, 30, and 50 mg/mL, 2 replicates for 10 mg/mL, and 1 analysis for 100 mg/mL; **b** 2 replicates for 5, 10, and 50 mg/mL and 1 analysis for 2, 20, and 30 mg/mL each; **c** 2 replicates for 5, 10, and 20 mg/mL and 1 analysis for 2, 30, and 50 mg/mL each

On the contrary, these measurements indicated a high sensitivity of RIfS (and 1-lambda reflectometry) with changes in the optical thickness of up to 1.4 nm for the binding of the FAK fraction with a molecular mass of 32.46 kDa; see Fig. 2a. For comparison, similar measurements with proteins such as IgG antibodies, ERα-LDB, and thrombin (app. 60–150 kDa) revealed changes in the optical thickness in RIfS of 0.2–2.5 nm [29, 39, 40].

### Monitoring kinase-inhibitor interactions with sensors

#### Single assays with one kinase-inhibitor pair

The interaction of the kinase FAK with STP immobilized on the transducer was followed with RIfS via the optical thickness recorded during the measurement using the protocol described in "Single assays" section. The binding curves for measurements with different FAK concentrations are shown in Fig. 2a. As in the first 200 s, only buffer was flushed over the sensor, no interactions with the inhibitors immobilized on the surface were expected, the optical thickness stayed constant. During the association phase (200–400 s), protein kinases were flushed over the sensor, interacting with the immobilized inhibitors and therefore changing the optical thickness. As non-specific interactions were not relevant (see "Optimization of the sensor assay" section), the strong increase in optical thickness is directly related to the specific interaction of FAK and STP. From the relatively large change in optical thickness, a strong affinity can be expected. The optical thickness during the binding phase increased with higher concentration of the FAK-solution as expected.

While flushing buffer after the association, some dissociation was expected, reasoned by the adjustment of the equilibrium between association and dissociation (400–1000 s). However, the sensograms in Fig. 2a did not show a decrease in optical thickness upon flushing with buffer during the dissociation phase at 400–1000 s. An exception is the measurement with the high FAK concentration of 50 µg/mL, at which additional weak interactions might have been present but lost during the rinsing step. For none of the experiments, the optical thickness returned to the baseline values recorded before binding kinases. This indicated a strong binding, well reflected in the inability to fully regenerate the surface. This necessitated single measurements with freshly prepared sensor surfaces (see "Regeneration of the sensor surfaces" section and S2).

With the success of titration experiments in RIfS, further work was conducted with 1-lambda reflectometry, which evaluates greyscale images recorded from the sensor area and the changes in the intensities in defined regions of interest (ROI) on the sensor. The advantage of 1-lambda reflectometry is the spatial resolution, which allows to immobilize different inhibitors as spots on the surface of one transducer (further discussed in "Single assays with one kinase-inhibitor pair" section). However, this necessitates a higher kinase consumption for each measurement. Different combinations of inhibitors and kinases were investigated: protein mix with STP (Fig. 2b) and with fragment1 (Fig. 2c). The results were similar to those in RIfS demonstrating the principal applicability of the method for different kinase-inhibitor pairs but also the strong and irreversible interaction on the sensor surface.

Further, we conducted binding inhibition assays. A constant concentration of the protein kinase (FAK or protein mix) was titrated with different concentrations of staurosporine in pre-incubations. Kinases that were not inhibited by the inhibitors in solution were then shown to bind to the sensor surface leading to a concentration-dependent change in optical thickness in RIfS or 1-lambda reflectometry (data not shown). This clearly demonstrates that the binding is specific and monitoring of the protein kinase-inhibitor interaction in the homogeneous phase is possible with the setup.

#### Kinetic evaluation

**Fig. 3:**
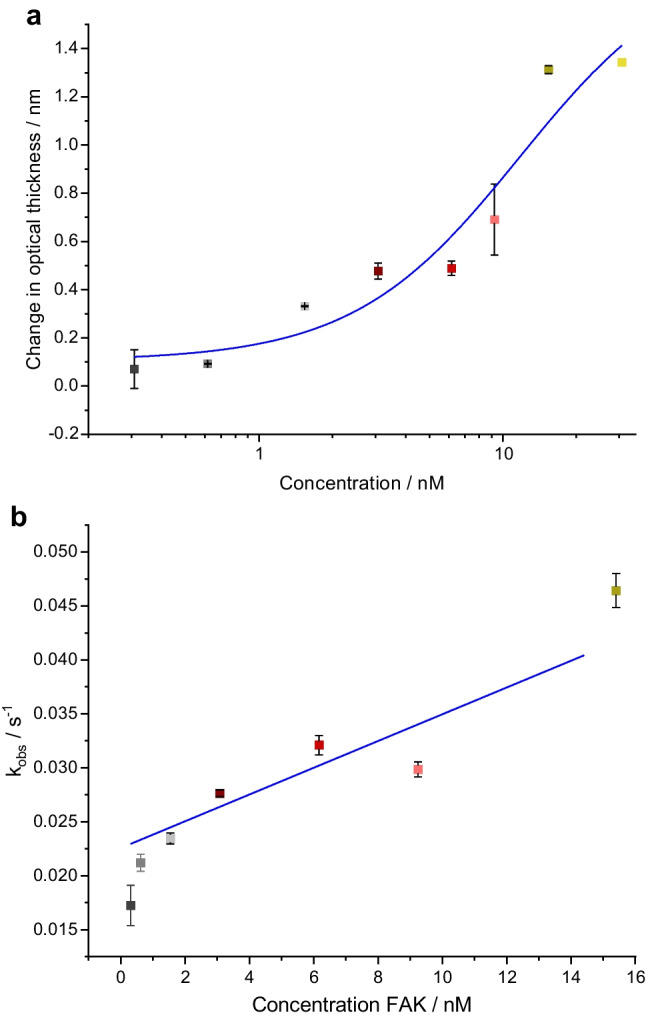
**a** Dose-response curve of the sensograms displayed in Fig. 2a. **b** Linear regression of the observable rate constants for the kinetic evaluation of the sensograms displayed in Fig. 2a. The colors of the data points are the same as in the sensograms in Fig. 2a. Error bars are based on the standard deviation of the optical thickness in three measurements

The binding affinity, defined by the dissociation constant *K*_*d*_ (affinity), and the binding kinetics with the association rate constant *k*_*a*_ and dissociation rate constant *k*_*d*_ are important parameters in drug discovery and helpful comparing results with other studies. The averaged change in optical thickness of the sensograms of similar kinase concentrations was plotted against the kinase concentration on a logarithmic scale. The law of mass action, describing the thermodynamics [41], shows that the asymptote at high concentrations describes the maximal system response, while the slope at the inflection point shows the system’s sensitivity [42, 43]. Figure 3a shows the sigmoidal form for this plot of the RIfS analyses shown in Fig. 2a expected with regard to the binding in the ATP pocket and steric limitations hindering secondary associations. The coefficient of determination of *R*^2^=0.932 for the fit function was high also with regard to the uncertainty introduced by the manual preparation of the transducer for single use and to the fact that saturation in the dose-response curve was not reached for all kinase-inhibitor pairs in the tested concentration range. Similar dose-response curves were obtained for the other two kinase-inhibitor pairs investigated with 1-lambda reflectometry (sensograms shown in Fig. 2b and c) (results not shown).

For further evaluation, an exponential fit was applied to the association phase of each sensogram to determine the observable rate constant *k*_obs_ (see "Kinetic evaluation" section, Eq. 1). In Fig. 3b, the observable rate constants *k*_obs_ were then plotted against the protein concentration (Eq. 2). The correlation was limited with *R*^2^=0.726 (FAK interacting with STP, see Fig. 2a), 0.94 (protein mix interacting with STP, see Fig. 2b), and 0.725 (protein mix interacting with fragment1, see Fig. 2c).

Assuming pseudo-first-order kinetics for the binding mechanism, we can use the linear regression in Fig. 3b, to estimate the rate constants *k*_*a*_ and *k*_*d*_ and with them *K*_*d*_. More complex models include possible rate-limiting steps after the first association when the precision of the assays is improved. For the protein mix, only a very rough estimate can be made assuming all proteins in the protein mix to be cAMP-dependent protein kinase (PKA). For the different pairs, we obtained the following:

- FAK-STP: *K*_*d*_=18.2 nM and kinetic data of *k*_*a*_=1.24 L µmol^−1^ s^−1^ and *k*_*d*_=22.5·10^−3^ s^−1^.
- Protein mix-STP: *K*_*d*_=205 nM and kinetic data of *k*_*a*_=24.4 L mmol^−1^ s^−1^ and *k*_*d*_=5·10^−3^ s^−1^.
- Protein mix-fragment1: *K*_*d*_=321 nM and kinetic data of *k*_*a*_=21.8 L mmol^−1^ s^−1^ and *k*_*d*_=7·10^−3^ s^−1^.

For comparison: for the interaction of FAK with STP, Kabir et al. determined similar dissociation constants *K*_*d*_ in a range of 10–100 nM via fluorescence microscopy [44]. For PKA interacting with STP, several studies reported *K*_*d*_ values in a range of 2–44 nM in different assay formats (enzyme assays, fluorescence polarization, phosphorylation assays) [17, 45–47]. These values were approximately one order of magnitude smaller than the dissociation constants determined in our study with 1-lambda reflectometry. These differences can mainly be explained by the fact that the protein mix does not only contain PKA.

For PKA interacting with various synthetic peptides in competition to ATP in a phosphotransferase assay, the results showed a broad range of 2 nM–500 µM for the dissociation constant [48] depending on the specific pair of protein kinase and inhibitors. The *K*_*d*_ value of 321 nM for the interaction of the protein mix, assumed as PKA, with fragment1 is well in the range of *K*_*d*_ values in a study by Glass et al. [48] for other inhibitors. Data for the interactions of PKA with this specific fragment1 cannot be found in literature. Such a fragment-based screening can be used to define molecular structures relevant for a specific binding in the ATP-binding pocket of a protein kinase to obtain high binding energies [49–53].

As rate constants are rarely determined, we could not find suitable reference values for the kinase-inhibitor pairs investigated here. Thus, we used microscale thermophoresis (MST) for comparison of the K_D_ value. MST monitors molecular interactions in the homogeneous phase. From a titration experiment with a constant concentration of fluorescently labeled STP and different concentrations of the protein mix or FAK (details in Section S4), we obtained dose-response curves of the normalized fluorescence plotted against the logarithmic kinase concentration (Figure S5). From this, we calculated the dissociation constants. These were higher than those for the sensor investigations: for FAK 796 nM (MST) vs. 18.2 nM (RIfS), for the protein mix 788 nM (MST) vs. 205 nM (1-lambda reflectometry). Differences are due to the labeling of the STP for MST analysis and inhibitor immobilization on the transducer for sensor investigations. Small changes in different regions in the molecular 3D structure, depending on the method of labeling, may already influence their interaction with the kinases. Furthermore, in general, stronger interactions of proteins with small molecules are observed, as shown for homogeneous vs. heterogeneous assays (MST vs RIfS) [54].

### Screening kinase-inhibitor interactions

1-Lambda reflectometry can be used in an array format and -combined with spotting techniques- record images of a full transducer surface. With RIfS, the principal applicability of the system including kinetic data evaluation was shown. The method was transferred to a screening format in order to simultaneously study different kinase-inhibitor pairs together with their affinities and kinetics.

#### Spotting

**Fig. 4:**
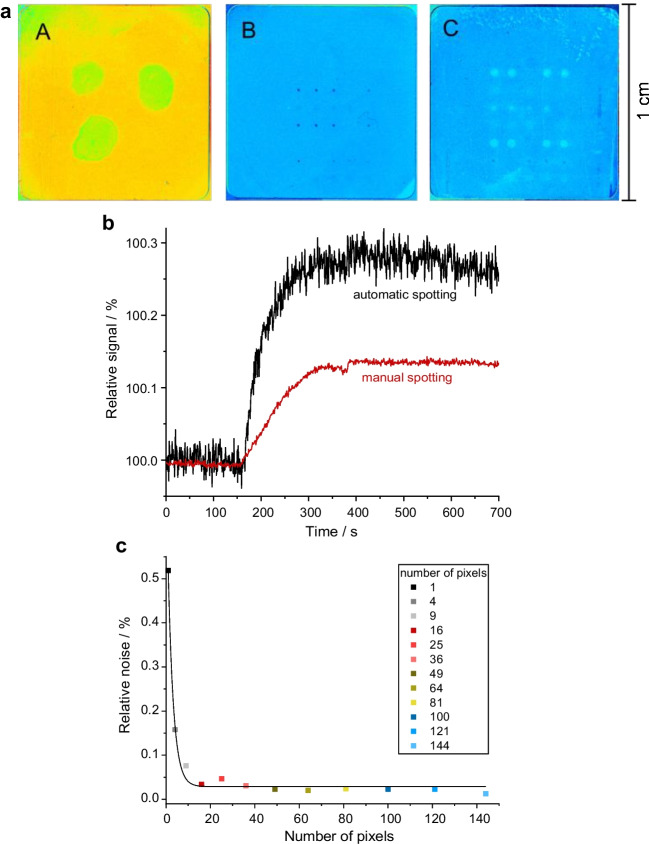
Inhibitor and kinase screening using 1-lambda reflectometry: **a** image of the measurement region of three transducer surfaces. (A) The inhibitor is spotted manually to the transducer. (B and C) The inhibitor is spotted automatically to the transducer via the spotter with different droplet volumes of approximately 1 nL (B) and approximately 4 nL (C). **b** Binding curves of a sample containing transforming growth factor β (TGFβ, 10 µg/mL) from manual spotting (red, ROI 121 pixels) and automatic spotting (black, ROI 4 pixels) on two different transducers modified with STP. **c** Dependence of the average value for the dark noise on the number of pixels used as the ROI, recorded from a baseline measurement with 1-lambda reflectometry (no kinase)

Two spotting methods were tested: manual spotting via pipetting and automatic spotting via an automatic spotter (details, see the “Surface chemistry” section). In Fig. 4, images of different transducer surfaces are shown after the association of kinases to spots of different inhibitors using 1-lambda reflectometry. Due to the higher volume of inhibitor solution used for the manual spotting, only three spots could be placed on the surface, leaving space for a region for reference required in 1-lambda reflectometry, see Fig. 4a, panel A. In Fig. 4a, panels B and C, automatically generated spots are visible in the images with their size depending on the spot volumes used (1 nL vs. 4 nL). Comparing the images in Fig. 4, it is clear that the automatic spotting is advantageous due to higher number of possible spots with a larger number of different inhibitors available for screening. In addition, precision is improved due to parallel experiments (here four spots per row and inhibitor).

In Fig. 4b, the sensograms of our screening experiments show that the automatic spotting evokes higher sensor signals but also higher noise.

We determined an optimal size of the ROI varying the pixel number used for evaluation between 1 and 144. The results in Fig. 4c show that the signal to noise ratio strongly depends on the spot size. With about 16 pixels, a low signal to noise ratio is reached. We chose ROIs for the evaluation of 121 pixels for the manually spotted transducers and 4–9 pixels for the automatically spotted ones in screening applications as a compromise between good signal to noise ratio and the number of spots applicable. 1-Lambda reflectometry with arrays containing over 5000 spots are already published, showing the high throughput achievable with this method [55].

**Fig. 5:**
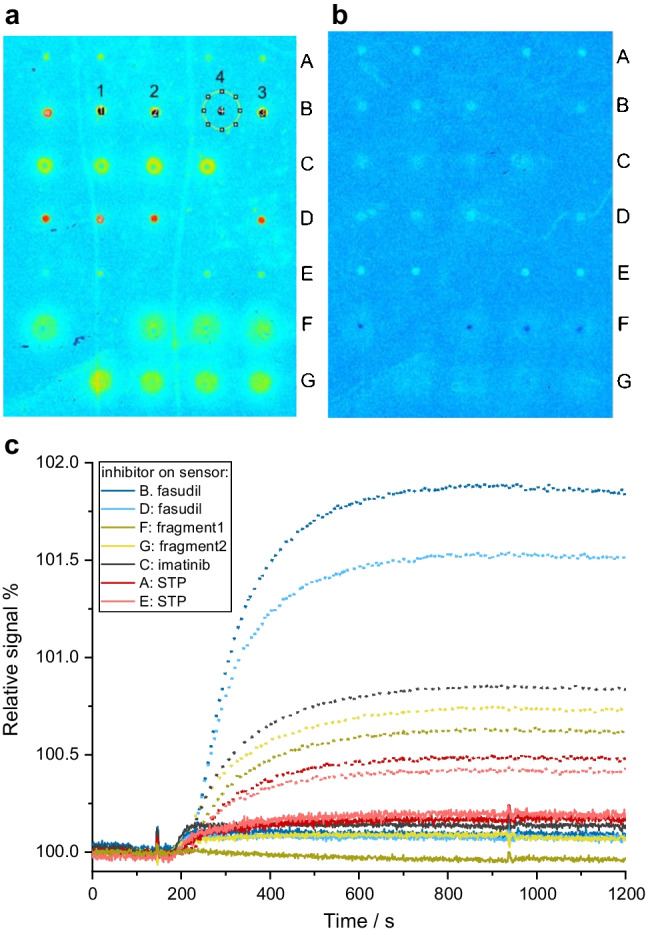
Image of the sensor array in 1-lambda reflectometry using 50 mg/mL of **a** protein mix and **b** FAK. Each row of the automatically produced spots comprises a different inhibitor: A+E, STP; B+D, fasudil; C, imatinib; F, fragment1; G, fragment2. Exemplarily, the ROIs for the evaluation are marked with 1–3, while 4 is the reference region in row B. **c** Sensograms of the screening, from the images displayed in **a** for the protein mix as sample (dotted lines) and **b** the FAK-sample (full lines). These binding curves can be used for kinetic data evaluation

The reference ROI was always defined larger than the inhibitor spots to reduce the influence of the noise by the reference as exemplarily shown in row A in Fig. 5a with ROIs 1–3 with inhibitor and ROI 4 void of inhibitor.

#### Inhibitor screening

With the setup and protocols chosen, inhibitor screening was performed with different inhibitors spotted in rows: STP, fasudil, imatinib, fragment1, and fragment2. One region per row was set as the reference ROI void of inhibitor. In Fig. 5a, the image of the transducer after the association of the protein mix (50 µg/mL) shows different changes in the color plot indicating that the kinase in the protein mix interacted differently with the different inhibitors. The strongest binding affinity can already be detected by naked eye via the strongest color change for fasudil, followed by imatinib > fragment2 > fragment1 > STP. The similarity of the spots within one row demonstrates a high repeatability of the assay (see Fig. 5a in rows B and D for fasudil and in rows A and E for STP).

Quantitative information on both binding kinetics and affinity was obtained from the sensograms in Fig. 5c, exemplarily recorded at one spot per row. For PKA in the protein mix, the signals decreased depending on the type of inhibitor immobilized on the transducer surface in the order of fasudil with 1.7% increase of the average relative signal followed by imatinib > fragment1 > fragment2 > STP (average increase in relative signal 0.45%). Clearly, a screening of kinase-inhibitor interactions is possible. Similarly, Fig. 5c shows inhibitor screening with FAK as the kinase. The order regarding the strength of the association of FAK as kinase sample with the different inhibitors is different to PKA and follows the order STP > imatinib > fasudil > fragment2 > fragment1, similarly to what would be inferred from visual inspection.

#### Kinase screening

We would like to emphasize that the methodology developed here can also be used for kinase screening. Comparing the results for Fig. 5a (protein mix as sample) and Fig. 5b (FAK as sample) using two different transducers, differences in the changes of the colors of each inhibitor spot are visible. As for the inhibitor screening, differences between the association of the proteins to the different inhibitors spotted are already visible from the images with brighter spots. This is further corroborated by the smaller changes in the relative signal for the samples containing FAK in the binding curves plotted in Fig. 5c.

The association on the imatinib spot significantly increased the relative signal by 0.84% with the protein mix but only 0.13% with FAK. For the other inhibitors spotted to the sensor surface, similar results were obtained. This shows that differences in the affinity for different kinase-inhibitor pairs can be visualized as well as kinetically evaluated via the binding curves. We demonstrate that both kinase and inhibitor screening are possible with our new methodology.

## Conclusion and outlook

In this work, we introduced two assays using direct optical transduction to monitor the interactions between protein kinases and inhibitors, which were covalently immobilized on the transducer surface. With RIfS, specific kinase-inhibitor pairs were addressed to not only provide thermodynamic data on the binding affinity but also data on the binding kinetics. This was possible with a low sample volume for direct assays and binding inhibition assays. Further improvements in the transducer modification are necessary to increase precision as regeneration of the sensor surfaces after analysis failed, necessitating single use of transducers.

With the optimized spotting of different inhibitors on one transducer, the advantages of 1-lambda reflectometry can be exploited: inhibitor and kinase screening are both possible with only one setup with different inhibitors spotted in rows. As images are recorded by a camera, a visual as well as a qualitative evaluation of the screening experiments is possible prior to in-depth evaluation. With this approach, it is possible to gain information on both thermodynamic and kinetic data. In the future, we will implement directed flow channels fed by different kinases in order to combine kinase and inhibitor screening in one measurement enabling a higher throughput in the screening [56]. Prospectively, a method is possible, with which hundreds to thousands of spots of covalently bound inhibitors could be screened for their interactions with various kinases with a microfluidic design of flow channels implemented [57]. These time-resolved measurements, requiring only a few minutes and approximately 200–400 µL of protein kinase solution, provide the kinetic data of association and dissociation rate constants as well as the affinity of the binding between the inhibitors and protein kinases via the dissociation constant.

In principle, the methodology may be transferred to the analysis of kinases in cell extracts, as biosensors were shown to be applicable to this matrix [58]. Then, mixture effects could also be studied [12]. In order to identify and purify kinases, we made first attempts of transferring the surface chemistry of the transducers to magnetic nanoparticles (see Section S5). This would enable specific or broad kinase capturing (depending on the inhibitors immobilized) from liquid samples to identify them in downstream LC-MS analysis but also to purify them for detailed investigations using RIfS as outlined in the review of Wurster et al. [7].

## Supplementary Information

Below is the link to the electronic supplementary material.

Supplementary file1 (DOCX 836 KB)
