# Supplementary material for: Reflectometric-based sensor arrays for the screening of kinase-inhibitor interactions and kinetic determination
Source: Anal Bioanal Chem. 2025 Feb 10;417(30):6811–22. doi: 10.1007/s00216-025-05770-x (PMC12680756; doi:10.1007/s00216-025-05770-x)
Supplement: Supplementary file 1 — Supplementary file1 (DOCX 836 KB) [file 216_2025_5770_MOESM1_ESM.docx]

Supporting Information to

Reflectometric-based sensor arrays for the screening of kinase-inhibitor interactions and kinetic determination

Viola Wurster^1^, Peter Fechner^1^, Günther Proll^2^, Rebecca Pamies-Cuberos^1^, Matthias Frech^3^, Jens Baumgärtner^3^, Antonia Malissa^4^, Martina Marchetti-Deschmann^4^, Natalia Ivleva^5^, Christoph Saal^3^, Sebastian Fuchs^3^, Sébastien Moniot^3^, Anja Göttsche^3^, Carolin Huhn^1^

^1^Eberhard Karls Universität Tübingen, Institute of Physical and Theoretical Chemistry, Auf der Morgenstelle 18, 72076 Tübingen, Germany

^2^Reutlingen University, Faculty of Life Science, Alteburgstraße 150, 72762 Reutlingen, Germany

^3^Merck KGaA, Frankfurter Straße 250, 64293 Darmstadt, Germany

^4^Technical University of Vienna, Institute of Chemical Technologies and Analytics, Getreidemarkt 9, 1060 Vienna, Austria

^5^Technical University of Munich, Institute of Water Chemistry, Lichtenbergstraße 4, 85748 Garching, Germany

## **S1 Kinetic Evaluation**

In Figure S1, the linear regression of the observable rate constants for the kinetic evaluation of the sensograms in Figure 2b and c (main text) are shown. 1-Lambda reflectometry was used to investigate the kinetics of the protein mix as a sample at increasing concentrations on transducers modified with STP or fragment1. The coefficient of determination was R²=0.73 for the linear fit when using STP as inhibitor (Figure S1a) but only 0.94 for transducers modified with fragment1 (Figure S1b). Clearly, the repeatability of the analysis is impaired as new transducers had to be prepared for every measurement due to the failure of the regeneration of the transducer surface.

Figure S1: Observable rate constants plotted against the concentration of the protein mix or FAK with linear regression to determine the kinetics of the binding process. Data source: (a) Figure 2b for the protein mix as sample on a transducer modified with STP and (b) Figure 2c for the protein mix as sample on a transducer modified with fragment1. Colors as in the sensograms in Figure 2.

## **S2 Irreversible Binding of Kinases**

S2.1 Protocols to Regenerate the Sensor Surface

The goal of the intense work on the regeneration of the sensor surface was to be able to reuse the sensors creating a more sustainable method and especially to enhance the comparability within measurement series. This requires to completely remove the kinases (or other proteins in case of non-specific binding) bound to the transducer surface after measurements. However, early experiments already showed that the regeneration was not straight-forward, so we tested several regeneration strategies using FAK in combination with transducers immobilized with STP. First, using RIfS, classically known regeneration media, such as acids and bases, chaotropes and detergents and various combinations of these solutions were investigated as summarized in Figure S2.


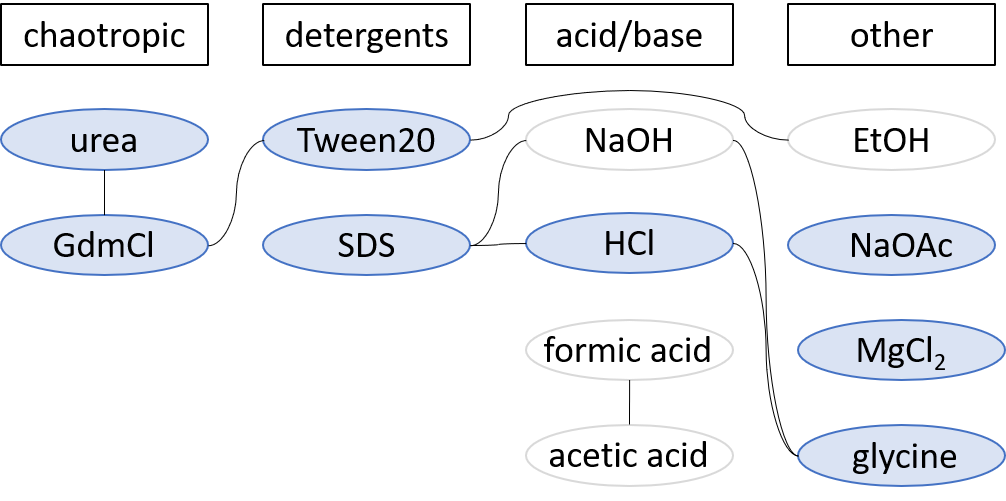


Figure S2: Chart of the first regeneration strategy tested, summarizing the regeneration media, which were tested alone, in combinations (as indicated with connections) or with repeated applications using twice the same medium (highlighted in blue).

Most of these regenerations reached an efficiency of only 45%-50%, only a few were more successful with an efficiency of 95%, determined by using the baseline before an analysis as a reference. This value was compared to the value of the optical thickness obtained after flushing the regeneration medium over the sensor. Unfortunately, in case of a successful regeneration, we observed that the kinase association with the inhibitor was impaired in later RIfS measurements with a smaller change in optical thickness compared to analyses with new sensors. This was likely due to irreversible changes in the surface chemistry.

As the classical approaches failed in regenerating the sensor surface, a second and broader strategy was followed which was based on cocktails of regeneration media developed by Andersson et al. for regeneration in surface plasmon resonance analyses (1). The cocktails of each class of regeneration media (acids, bases, chaotropes, etc…) were indicated with upper-case letters (A, B, C, etc.). These cocktails were expected to improve the regenerative effects compared to the use of a regeneration medium separately. Table S1 shows the cocktail mixtures tested together with the efficiency of the regeneration determined by the ratio between endline and baseline of sensor measurements. Good regeneration efficiencies of 97% were observed with the cocktail mixture DUw combining detergents and non-polar solvents. Unfortunately, subsequent RIfS analyses with FAK were not reproducible, so the regeneration impaired as a reuse of transducers was not possible.

Table S1: Regeneration cocktail mixtures according to Andersson et al. (1) tested as a second strategy for FAK regeneration on transducers modified with STP. The efficiency of each regeneration cocktail is indicated in %. Cocktails that impaired a following protein kinase association after the particular regeneration are highlighted in bold. The media were: A: acids (formic acid, H_3_PO_4_, malonic acid, oxalic acid - each 0.15 M – pH 5 (with 1 M NaOH)), B: bases (ethanolamine, glycine, Na_3_PO_4_, piperazine - each 0.2 M – pH 9.2 (with 1 M HCl)), C: chelators (Na_2_−EDTA – 20 mM), D: detergents (CHAPS, Triton X-100 - each 0.3% (w/w), Tween20, Tween80, Zwittergent3-12 - each 0.3% (v/v)), I: ionic compounds (guanidine hydrochloride (1.83 M), KSCN (0.46 M), MgCl_2_ (1.83 M), urea (0.92 M)), U: non-polar, water-soluble compounds (1-butanol, acetonitrile, DMSO, ethanol, formamide - in equal amounts). The cocktail mixtures are named with a three-letter code, such as DUw (one part cocktail D + one part cocktail U + one part water).

| **Cocktail mixtures and regeneration effciency** | | | | |
| --- | --- | --- | --- | --- |
| Aww  - | ACw  30% | ADw  67% | AIw  21% | AUw  - |
| Bww  17% | BAw  18% | BCw  19% | BDw  75% | BDU  30% |
| Cww  4% | DCw  41% | Dww  6% | **DUw**  **97%** |  |
| Iww  20% | ICw  9% | IDw  23% | UCw  68% | UUw  71% |

A possible reason for the low regeneration is a dense layer of protein present on the transducer surface, which may have inhibited the action of the regeneration medium. Therefore, in a third strategy, we investigated if a tryptic digestion of the proteins supported the regeneration. However, the use of trypsin-EDTA followed by regeneration revealed regeneration efficiencies of about 45-60%. With the failure of sufficient regeneration, we decided to use new sensors for each measurement.

### S2.2 Surface Analysis

To better understand the causes for the strong interaction of the kinases with the transducer surface, we used the surface analytical techniques (i) attenuated total reflection infrared spectroscopy (ATR-IR spectroscopy), (ii) matrix-assisted laser desorption/ionization coupled to time of flight mass spectrometry (MALDI-TOF-MS) and (iii) Raman microscopy. All measurements were performed on RIfS transducers modified with AMD and STP. FAK that was associated via a RIfS experiment on the surface, verified by changes in the optical thickness of approximately 1 nm, was used as a sample and analyzed without any regeneration steps. Transducers modified with AMD and STP were used as reference samples.

MALDI-TOF-MS investigations were performed on a target plate constructed in-house from ITO slides and copper tape to ensure sufficient conductivity. The MALDI process was optimized using standard peptide target plates and sinapinic acid as matrix with the MALDI spectrum shown in Figure S3a (red trace). However, signals for FAK were not observed on the RIfS transducers, see Figure S3a (black trace). The assumed low FAK concentration from sensor measurements in combination with a too low conductivity provided by the target plates constructed in-house are likely reasons. The presence of FAK on the transducers after RIfS experiments could not be proven by MALDI-TOF-MS.

In ATR-IR experiments, no differences in the spectra were visible comparing transducers with and without FAK bound, as seen in Figure S3b, especially not in the range of 1450-1750 cm^-1^ from the protein’s amide moieties. As for MALDI, the sensitivity was insufficient to detect FAK in a monolayer on the transducer surface. Similarly, Raman microscopy failed in detecting kinases, see Figure S3c.

**a**


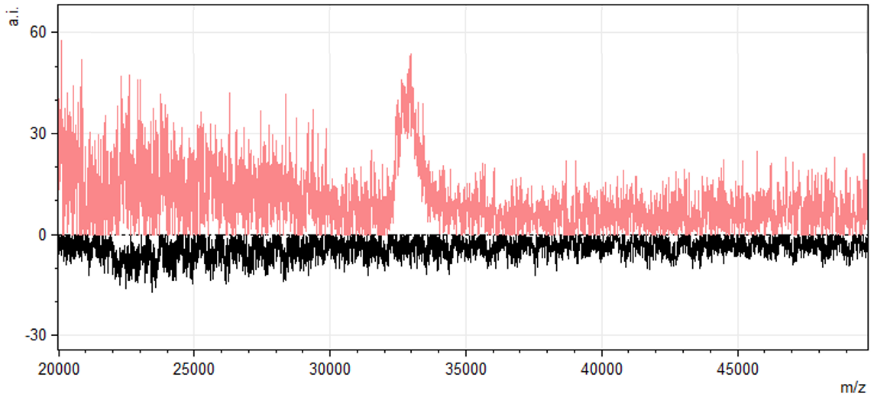

Figure S3: Results of the surface analysis of FAK present on the transducer from RIfS measurement. Transducers modified with AMD and STP were used with and without FAK association. a) MALDI-TOF mass spectra of FAK on a standard protein target plate as reference (red) and of a RIfS transducer with FAK associated (black). b) ATR-IR spectra and c) Raman spectra of transducers with and without FAK association.

### **S3 Docking Simulations**

Docking simulations were performed with the open source softwares AutoDock 1.5.6, AutoDock Vina 1.5.6 (Scripps Research, La Jolla, Canada), and PyMOL 3.0 (DeLano Scientific LLC, Schrädinger) (2, 3). The inhibitor molecules were drawn in 3D with ChemSketch, freeware version (ACD/Labs, Toronto, Canada). The crystal structures of the protein kinases were downloaded from the Protein Data Base (wwPDB Foundation, open access database) (4). During the simulations, the protein was treated as rigid structure. As output, the nine interactions sites of the inhibitor on the protein structure with the highest simulated free binding energies were shown. Both the interaction sites over the whole protein structure and inside the ATP-binding pocket were considered.

The inhibitors used in this docking simulation were chosen being approved drugs such as fasudil and imatinib. Bisindolylmaleimide X (BisX) is a molecule very similar to STP and thus ideal to study the effect of small changes in the molecular structure. BisX is an inhibitor for protein kinase C (5).

Simulations using the whole protein structure showed similar results for different protein kinases and different inhibitors: interactions were simulated to take place inside and outside the ATP-binding pocket, for an example, see Figure S4a for FAK and staurosporine. High binding energies of -9 to -7 kcal/mol were recorded for the interaction of PKA and FAK with staurosporine as well as imatinib (see Figure S4), similar to literature findings (2, 3, 6).


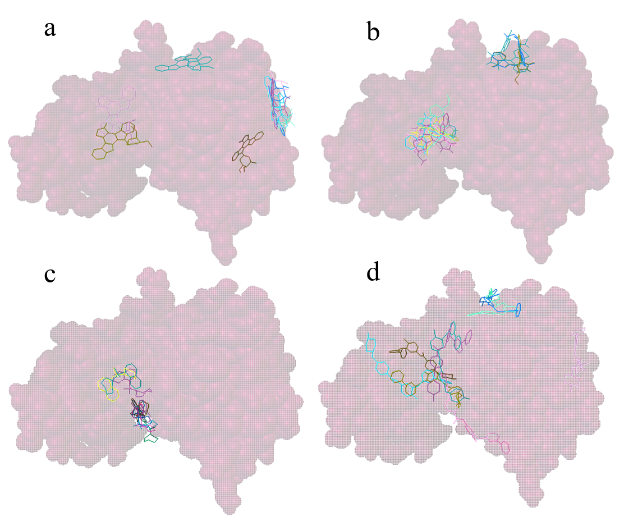


Figure S4: Results of the docking simulations of a) STP, b) BisX, c) fasudil and d) imatinib on FAK (PDB ID: 2AEH) over the whole protein structure. The output for the nine interaction sites with the highest calculated free binding energies are displayed. The simulated interaction sites are located over the whole protein kinase structure, not only in the ATP-binding pocket (2, 3).

## **S4 Microscale Thermophoresis**

Microscale Thermophoresis (MST) measurements were performed with different combinations of kinases and inhibitors. Figure S5a exemplarily shows the logarithmic dose-response curve of a direct MST experiment using the protein mix as the kinase sample over a large concentration range of 80 pM to 2 µM and fluorescently labeled STP (STP-Red, Perkin Elmer, Massachusetts, USA) with a constant concentration of 40 µM. Thus, we covered ratios from 50:1 to 2:10^6^ of inhibitor:kinase. Thermodynamic data of our MST measurements were found to be up to two orders of magnitude higher (K_d_=600-800 nM) than reported in other studies (7-11) and also compared to the sensor investigations of this work. Clearly, the effect of the multiple strong interaction sites of the inhibitors to the protein structures, as shown with the docking simulations in Section S3 necessitates further research.

Figure S5: a) Results of the MST measurements with fluorescently labeled STP at a concentration of 40 µM interacting with the protein mix titrated in a concentration range of 80 pM to 2 µM. For a better comparability, the fluorescence intensity obtained during the measurement was normalized to the fluorescence in each capillary recorded prior to the start of the experiment. The dose-response curve was fitted revealing a coefficient of determination of R²=0.981. b), c) Dose-response curves of a competitive MST experiment using the kinase FAK and fluorescently labeled STP in competition with rising concentrations of b) BisX (R²=0.990) and c) imatinib (R²=0.985).

In competitive MST assays, fluorescently labeled STP (40 µM) was first allowed to form a complex with FAK (40 nM) at constant concentrations. Prior to the analysis, the inhibitors BisX, imatinib or fasudil were titrated to record the dose-response curves. For BisX, the slope was inversed compared to the one of the direct assays, compare Figure S5a and b, as expected for a competitive assay. However, when titrating imatinib (Figure S5c) or fasudil (data not shown), the dose-response curves were similar to the one of a direct assay. Despite identical experimental conditions for all competitive MST assays different inhibitors inhibitor caused differences in the slope of the dose-response curves (12-14).

We hypothesize the differences in the dose-response curves to be due to different bindings sites present on the kinases: The docking simulations of FAK with different inhibitors (see Figure S4) showed that BisX can bind to FAK at two sites, which showed high binding energies also for STP in the docking simulation (see Figure S4a and b). Thus, for both sites competitive interactions can be expected, so that the titration of BisX forms BisX-FAK complexes. Accordingly, some STP-molecules are released. This changes the diffusion of the fluorescently labeled molecules and can thus be detected by MST with the expected dose-response curve. In contrast, both fasudil and imatinib showed interaction sites on FAK, that were not relevant in the interaction with STP (see Figure S4c and d). By titrating these inhibitors to the STP-FAK complex, the inhibitors may bind to these additional sites on the protein kinase. This behavior results in dose-response curves similar to the ones of the direct assays. These multiple bindings of inhibitors on the protein kinase, shown theoretically in the docking simulations and corroborated experimentally by the competitive MST experiments confirm the hypothesis, that the protein kinases can interact with several inhibitors on the transducer surface, creating a strong binding, which is robust against diverse regeneration experiments.

### **S5 Kinase Capturing by Nanoparticles with a Surface Chemistry Similar to the Biosensors**

We transferred the surface chemistry to magnetic nanoparticles to covalently bind STP to the particle surface. The magnetic nanoparticles, nanomag-CLD, with a diameter of 500 nm and a surface coating with PEG-COOH were purchased from micromod Partikeltechnologie, Rostock, Germany. The dextran-coated nanoparticles (500 µL), functionalized with carboxyl groups, were modified with the coupling protocol provided by the vendor. Briefly, the particles were activated by an NHS EDC coupling protocol in MES buffer on a vibrating table at 400 rpm for 45 minutes at ambient temperature. The activated particles were resuspended in PBS buffer (0.15 M NaCl and 10 mM KH_2_PO_4_, adjusted to pH 7.4) and STP was immobilized using a solution of 1 mg/mL in DMSO and a volume ratio of 1:3.3 (STP solution:PBS buffer suspension with nanoparticles) for 3 hours at 400 rpm at ambient temperature. The particles were washed with PBS buffer, resuspended in 200 µL PBS buffer and stored at 4°C until use.

Kinase solutions in different concentrations were added to a homogenized suspension of modified magnetic nanoparticles at a volume ratio of 1:10 (protein stock solution:nanoparticle suspension). This mixture was incubated on a vibrating table for 15 minutes at 400 rpm at ambient temperature. The particles were magnetically captured and the concentration of protein in the supernatant was quantified via its tryptophan fluorescence at 345 nm. The fluorescence intensity decreased in the supernatant after the removal of the nanoparticles with kinases bound to them compared to the original protein solution (data not shown). These first experiments show that the kinases FAK and TGFβ could well be captured from protein solutions by interaction with the inhibitor and magnetic capturing of the nanoparticles.

### **S6 References**

1. Andersson K, Haemaelaeinen M, Malmqvist M. Identification and optimization of regeneration conditions for affinity-based biosensor assays. A multivariate cocktail approach. Anal Chem. 1999;71:2475-81.

2. Trott O, Olson A. AutoDock Vina: Improving the speed and accuracy of docking with a new scoring function, efficient optimization, and multithreading. J Comput Chem. 2010;31:455-61.

3. Guterres H, Im W. Improving protein-ligand docking results with high-throughput molecular dynamics simulations. J Chem Inf Model. 2020;60:2189-98.

4. Consortium TU. UniProt: The universial protein knowledgebase in 2023. Nucleic Acids Res. 2023;51:D523-D31.

5. Brehmer D, Godl K, Zech B, Wissing J, Daub H. Proteome-wide identification of cellular targets affected by bisindolylmaleimide-type protein kinase C inhibitors. Mol Cell Prot. 2004;3(5):490-500.

6. Cavasotto C, Abagyan R. Protein flexibility in ligand docking and virtual screening to protein kinases. J Mol Biol. 2004;337:209-25.

7. Meggio F, Deana A, Ruzzene M, Brunati A, Cesaro L, Guerra B, et al. Different susceptibility of protein kinases to staurosporine inhibition. Eur J Biochem. 1995;234:317-22.

8. Tamaoki T, Nakano H. Potent and specific inhibitors of protein kinase C of microbial origin. Biotech. 1990;8:732-5.

9. Herbert J, Seban E, Maffrand J. Characterization of specific binding sites for [3H]-staurosporine on various protein kinases. BBRC. 1990;171:189-95.

10. Breitenlechner C, Gassel M, Hidaka H, Kinzel V, Huber R, Engh R, et al. Protein kinase A in complex with Rho-kinase inhibitors Y-27632, Fasudil, and H-1152P: Structural basis of selectivity. Struct. 2003;11:1595-607.

11. Bjorge J, Kudlwo J, Paterson A. Inhibition of stimulus-dependent epidermal growth factor receptor and transforming growth factor-alpha mRNA accumulation by the protein kinase C inhibitor staurosporine. FEBS Lett. 1989;243:404-8.

12. Seidel S, Dijkman P, Lea W, van den Bogaart G, Jerabek-Willemsen M, Lazic A, et al. Microscale thermophoresis quantifies biomolecular interactions under previously challenging conditions. Methods. 2013;59:301-15.

13. Lippok S, Seidel S, Duhr S, Uhland K, Holthoff H, Jenne D, et al. Direct detection of antibody concentration and affinity in human serum using microscale thermophoresis. Anal Chem. 2012;84:3523-30.

14. Patniak S, Zheng W, Choi J, Motabar O, Southall N, Westbroek W, et al. Discovery, structure−activity relationship, and biological evaluation of noninhibitory small molecule chaperones of glucocerebrosidase. J Med Chem. 2012;55:5734-7548.
